# A cross-sectional comparative study of gut bacterial community of Indian and Finnish children

**DOI:** 10.1038/s41598-017-11215-y

**Published:** 2017-09-05

**Authors:** Shreyas V. Kumbhare, Himanshu Kumar, Somak P. Chowdhury, Dhiraj P. Dhotre, Akihito Endo, Jaana Mättö, Arthur C. Ouwehand, Samuli Rautava, Ruchi Joshi, Nitinkumar P. Patil, Ravindra H. Patil, Erika Isolauri, Ashish R. Bavdekar, Seppo Salminen, Yogesh S. Shouche

**Affiliations:** 1 Department of Microbiology, R.C. Patel Arts, Science, and Commerce College, Shirpur, Dist. Dhule, Maharashtra 425405 India; 2 National Centre for Cell Science, Savitribai Phule University of Pune campus, Ganeshkhind, Pune, Maharashtra 411007 India; 3 Functional Foods Forum, Faculty of medicine, University of Turku, Turku, 20520 Finland; 4 Max Planck Institute for Biogeochemisty, Jena, 07747 Germany; 5 Department of Food and Cosmetic Science, Tokyo University of Agriculture, Hokkaido, Japan; 6 Finnish Red Cross Blood Service, Kivihaantie 7, Helsinki, 00310 Finland; 7 Active Nutrition, DuPont Nutrition & Health, Kantvik, 02460 Finland; 8 Department of Paediatrics, University of Turku, Turku, 20520 Finland; 9 King Edward Memorial hospital research centre, Pune, Maharashtra 411011 India; 10 Department of Microbiology, Smt. Chandibai Himathmal Mansukhani College, Ulhasnagar, Thane, Maharashtra 421003 India

## Abstract

The human gut microbiome plays a crucial role in the compositional development of gut microbiota. Though well documented in western pediatrics population, little is known about how various host conditions affect populations in different geographic locations such as the Indian subcontinent. Given the impact of distinct environmental conditions, our study assess the gut bacterial diversity of a small cohort of Indian and Finnish children and investigated the influence of *FUT2* secretor status and birth mode on the gut microbiome of these populations. Using multiple profiling techniques, we show that the gut bacterial community structure in 13–14-year-old Indian (n = 47) and Finnish (n = 52) children differs significantly. Specifically, Finnish children possessed higher *Blautia* and *Bifidobacterium*, while genera *Prevotella* and *Megasphaera* were predominant in Indian children. Our study also demonstrates a strong influence of *FUT2* and birth mode variants on specific gut bacterial taxa, influence of which was noticed to differ between the two populations under study.

## Introduction

Microbial communities associated with the human gut respond and interact with host immune and digestive functions, thus contributing greatly to the overall well-being of the human body^1–3^. Previous studies have shown that such microbe-human interactions begin as early as during the gestation period and evolve in a major way at birth, followed by multiple environmental challenges. Host genetics, dietary habits and lifestyle play key roles in compositional development of the microbiome^2, 4–15^. Conditions such as malnutrition in the early stages of life are known to delay the maturation of gut microbiome and thus to hamper the development of metabolic functions in the gut^1, 16, 17^. Additionally, the precocious alterations in the gut microbiome are known to be the major contributors for the underlying mechanism of early age obesity^18–20^. These alterations can lead to long-term consequences with increased risk of diseases in the later life. In summary, understanding the implications of development trajectory of gut microbiota in health and disease is necessary to determine the influence of such elements.

There is growing evidence to suggest the association of host genotype with the microbial community structure. A well-characterized association of this type in western population is *FUT2* gene polymorphism. This gene encodes for an enzyme fucosyltransferase-2 (α-1,2-fucosyl transferase) which is involved in synthesis of the ABO antigen^21, 22^. Single Nucleotide Polymorphism (SNP) in the exonic region of this gene is also known to be associated with certain gastrointestinal diseases such as Inflammatory Bowel Disease (IBD) and Crohn’s disease^23–25^. It is evident from few studies that this molecule is also secreted from mucosal surfaces such as the intestinal lining and may serve as the source of glycans and adhesion site to gut microbes like lactic acid bacteria^26–28^. Very few attempts have been made to assess the association of this polymorphism with specific gut bacterial taxa^22, 26, 28, 29^ and to our knowledge, no efforts have been undertaken to compare this effect in different geographical settings.

Furthermore, the early vaginal contact during normal delivery of an individual is a major source of microbial diversity followed by the microbiome acquired from the breast milk^30–32^. Vaginal secretions ensure inoculation of the fetus at birth with specific microbial groups which carry out important functions necessary for normal development of the neonate^11, 33, 34^. Previous studies indicate that the neonatal gut microbiome is strongly associated with the mode of delivery and impacts the immune system development of the neonate^9, 35, 36^. The impetuous microbial disturbances introduced due to an altered mode of delivery are associated with increased risk of diseases^37, 38^. Overall, the association of practices in the early life of infants, with the gut microbial alterations is now clear from the extensive studies carried out in the western cohorts; however, there is scarce information available about such influences in the Indian population.

Investigations worldwide have suggested differences in the gut microbial composition between distinct geographical locations or populations^39, 40^. Also, international cohort studies suggest that inter-country variation in gut microbiota composition is significantly greater than inter-personal variations^41–43^. Collectively, though well documented, what remains unknown is whether the effect of these host elements and environmental challenges that cause microbial composition shifts in the western population also hold true in a heterogeneous and ethnically distinct non-western population. The present work aims at comparing the gut microbiota of 13-14-year-old Indian and Finnish children in a pilot study cohort, and also to assess the influence of *FUT2* polymorphism and mode of delivery on the gut microbial composition of these populations.

## Results

### *FUT2* secretor status of Indian and Finnish children

Genotyping of *FUT2* SNP rs601338 (W143X) revealed the secretor status of 99 subjects (52 Finnish and 47 Indian children). The study cohort included 45 and 40 secretors from Finnish and Indian population respectively, and 7 non-secretors each, from both the population. Secretor state GG/GA and non-secretor state AA were observed in both cohorts (Table 1).

**Table 1:** Demographic and genotypic characters of the subjects.

Description	Finnish	Indian
**1. Sex (%)**		
Male	27 (51.92%)	22 (46.80%)
Female	25 (48.07%)	25 (53.19%)
Total	52	47
**2. Age (Mean ± SD)**	13.73 ± 0.23	13.37 ± 0.46
**3. Mode of delivery (%)**		
Vaginal	44 (84.6%)	33 (70.2%)
Cesarean	8 (15.3%)	14 (29.8%)
**4. Secretor description (%)**		
Secretor	45 (86.53%)	40 (85.10%)
Non secretor	7 (13.46%)	7 (14.89%)
**5. Genotype (%)**		
AA	7 (13.46%)	7 (14.89%)
GA	23 (44.23%)	18 (38.29%)
GG	22 (42.30%)	21 (44.68%)

The table describes demographic characteristics of the Finnish and Indian cohort along with the *FUT2* secretor status as determined by genotyping and information about the birth mode of subjects.

### Targeted quantification of specific microorganisms in the human gut

**Figure 1:**
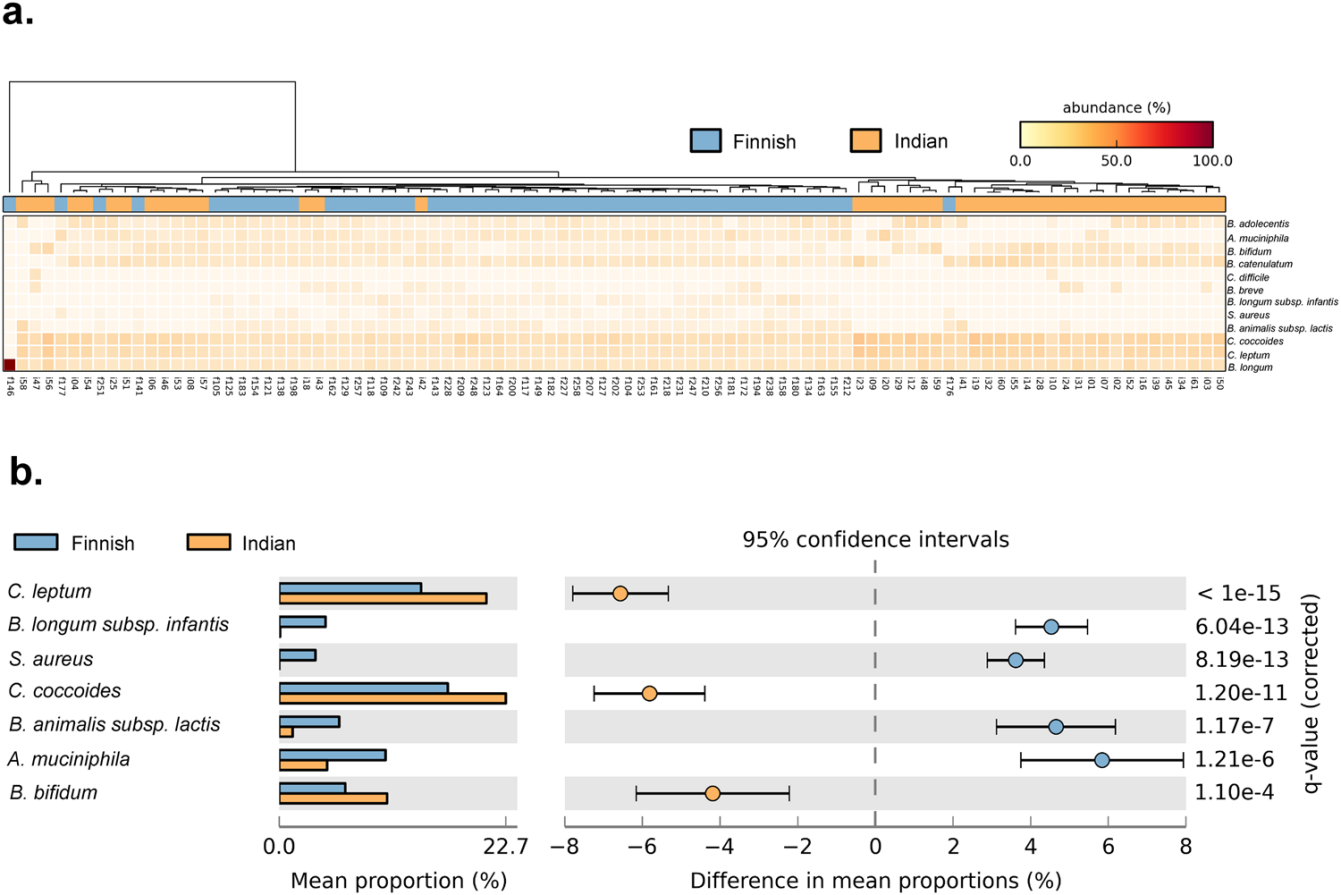
Real time quantification of bacterial species. (**a**) Heat Map is illustrating the extent of differences in the abundance of targeted bacterial species across samples from a Finnish and Indian population. (**b**) Extended bar plot is representing the differential abundance of significantly different bacterial species based on real time quantification data.

qPCR-based quantifications of 12 bacterial species (Supplementary Table S1) were carried out to determine their absolute abundance in the gut of Indian and Finnish children. Closer investigation of the heat map analysis demonstrated an overall higher abundance of bacterial species from *Clostridium* group in Indian children along with *Bifidobacterium bifidum* and *Bifidobacterium longum* group (Fig. 1a). Extended bar plot analysis revealed significant differences in 7 out of 12 bacterial species (Fig. 1b), with *Bifidobacterium longum* subsp. *infantis* and *Bifidobacterium animalis* subsp. *lactis* being significantly higher in Finnish children (Welch’s *t*-test, q = <0.001) along with *Staphylococcus aureus* (Welch’s *t*-test, q = <0.001) and *Akkermansia muciniphila* (Welch’s *t*-test, q = <0.001). While, *Bifidobacterium bifidum*, *Clostridium coccoides* group, and *Clostridium leptum* were significantly higher in Indian children (Welch’s *t*-test, q = <0.001). Distinct clades of Indian and Finnish samples in the Unweighted Pair Group Method with Arithmetic Mean (UPGMA) based dendrogram analysis also indicates differences in gut microbiota between these populations (See Fig. 1a). Principal component analysis showed distinct clustering of Indian and Finnish samples, while it also revealed that most of these bacterial taxa contributed to explaining the uniqueness of Finnish cohort, while only species from *Clostridium* group were found to be driving component for distinct clustering of the Indian cohort (Supplementary Fig. S1).

### Differences in gut microbial community structure between Indian and Finnish children

**Figure 2:**
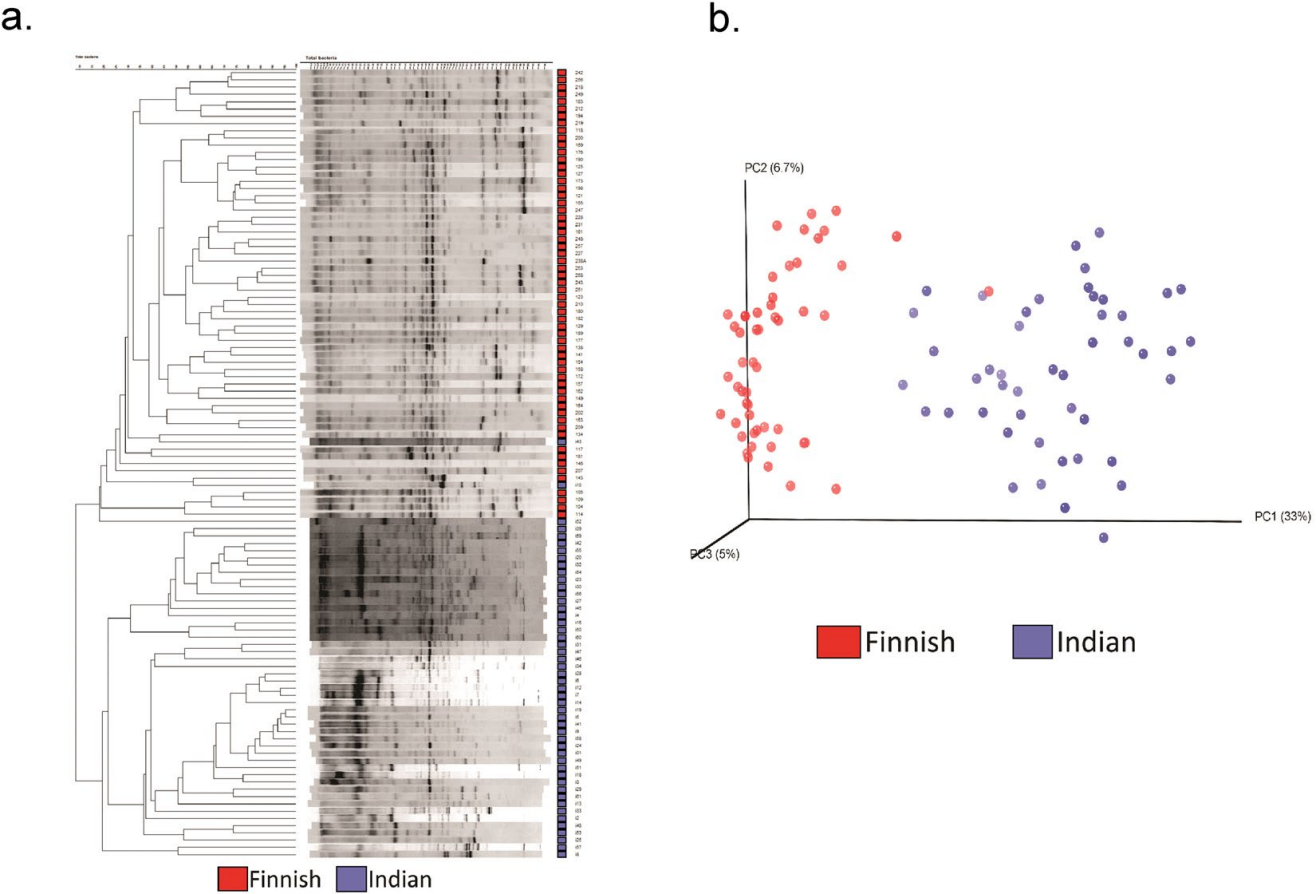
DGGE based diversity analysis. (**a**) UPGMA Cluster analysis based on PCR-DGGE analysis fingerprints from all samples. Finnish samples (red) and Indian samples (purple). (**b**) PCoA plot using Euclidean distances based on the prevalence of bands, representing differences in diversity between two populations: Finnish samples (red) and Indian samples (purple).

**Figure 3:**
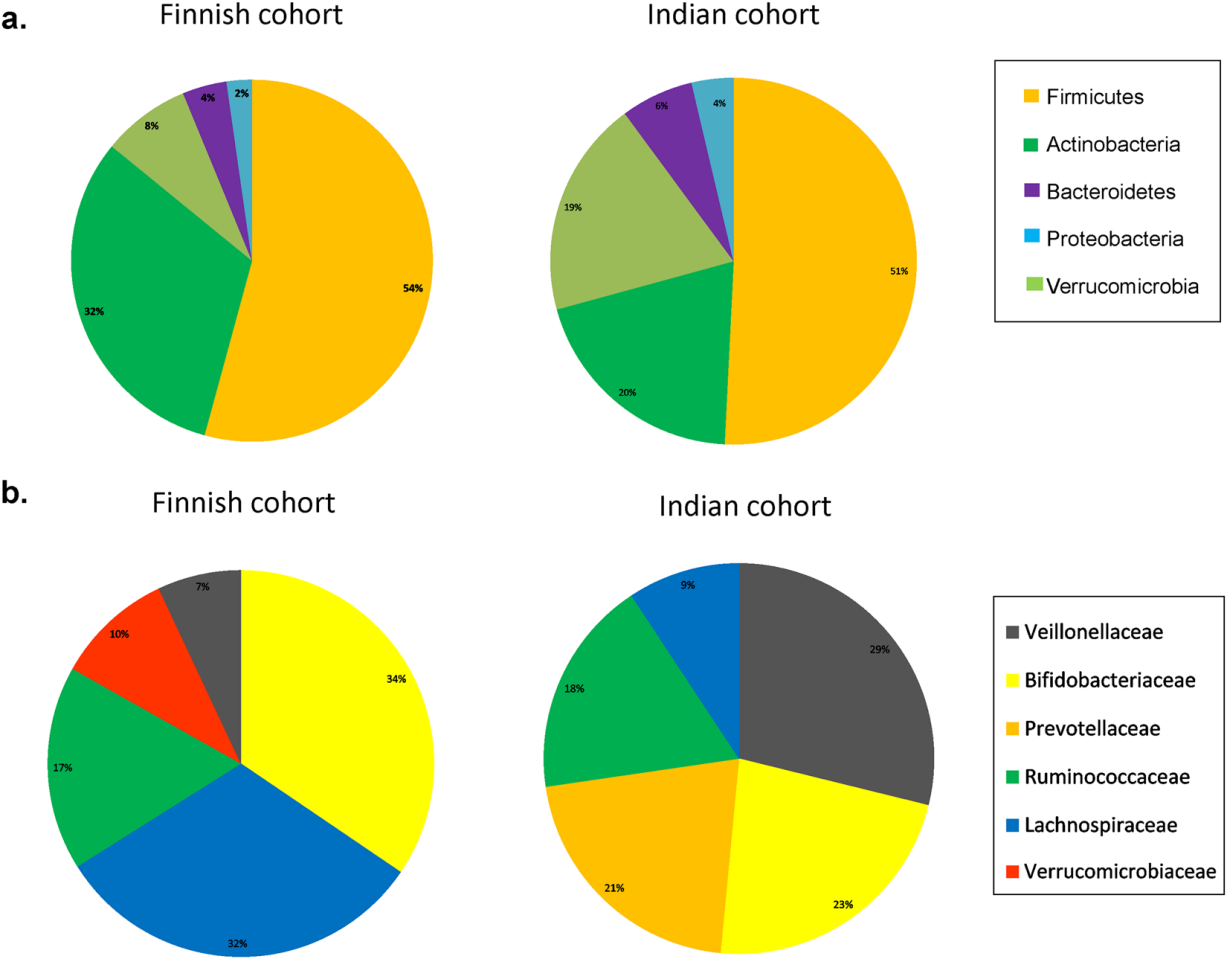
Gut bacterial community composition. (**a**) Phylum level distribution of dominant gut bacterial groups within Indian and Finnish children. (**b**) Family level distribution of dominant gut bacterial groups within Indian and Finnish children.

PCR-DGGE was carried out using the total DNA extracted from fecal samples resulting in obtaining 99 individual bacterial community fingerprints (standard strains used are listed in Supplementary Table S2). Further, this fingerprint-based dendrogram analysis demonstrated distinct clustering of two populations under study (Fig. 2a). Additionally, computer-assisted data matrix (distance matrix) was generated based on microbial community signature, to estimate various distances between samples and construct ordination plots using Principal Coordinate Analysis (PCoA). Ordination analysis performed on the DGGE data also revealed distinct clustering of Indian and Finnish subjects based on the prevalence of bands (Fig. 2b). Further, microbial diversity analysis performed using High Throughput Sequencing (HTS) suggested the presence of Firmicutes, Actinobacteria, Bacteroidetes, Proteobacteria and Verrucomicrobia as the most dominant phyla in both Finnish and Indian children (Fig. 3a). Bacterial composition at family level was dominated by *Bifidobacteriaceae* (34%) and *Lachnospiraceae* (32%) in Finnish subjects. While *Veillonellaceae* (29%) group was found to be the most dominant bacterial family in Indian children followed by *Bifidobacteriaceae* (23%) and *Prevotellaceae* (21%) (See Fig. 3b). Genera such as *Bifidobacterium* and *Blautia* were dominant in Finnish children, while the gut bacterial composition of Indian children was dominated by *Prevotella* and *Megasphaera*.

**Figure 4:**
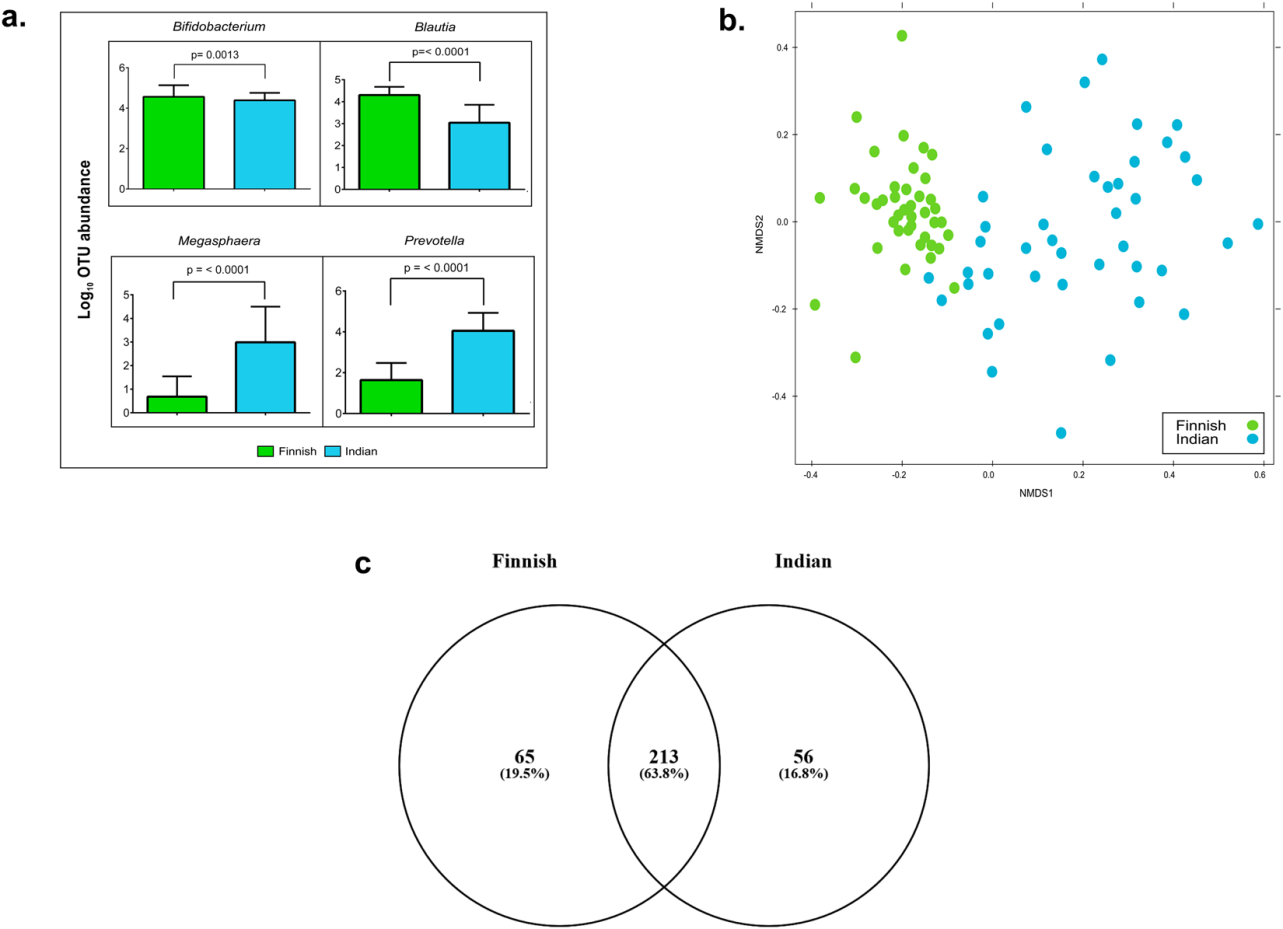
Beta diversity analysis. (**a**) Box plot illustrating differences in relative abundances of key contributors (most dominant) of bacterial community between Indian (blue) and Finnish children (green). (**b**) Beta diversity analysis using NMDS plot based on OTU level differences between Finnish (green) and Indian (blue) children (**c**) Venn diagram representing shared and unique bacterial genera between the gut bacterial community of Finnish and Indian children.

Variation in the gut microbial composition was observed in between Indian and Finnish children by both PCR-DGGE and 16S rRNA gene-based amplicon sequencing. Alpha diversity analysis showed that Finnish children harbored a marginally more diverse bacterial community than Indian children as indicated by the diversity measures such as observed species and Shannon index (Supplementary Table S3). Furthermore, phyla Actinobacteria (Welch’s *t*-test, q = <0.001), Bacteroidetes (Welch’s *t*-test, q = <0.001) and TM7 (Welch’s *t*-test, q = 0.026) were differentially abundant in between these two populations (Supplementary Fig. S2a). Apart from few bacterial families (Supplementary Fig. S2b), genera such as *Bifidobacterium*, *Blautia*, *Megaspahera*, *Prevotella*, *Ruminococcus*, *Dorea*, and *Coprococcus* were found to be differentially abundant in these two populations (Supplementary Fig. S2c). Of note, bacteria belonging to genera *Bifidobacterium* (Mann-Whitney U-test, p = <0.0013) and *Blautia* (Mann-Whitney U-test, p = <0.001) were significantly higher in Finnish children, while Indian children displayed significantly higher abundance of *Prevotella* (Mann-Whitney U-test, p = <0.001) and *Megasphaera* (Mann-Whitney U-test, p = <0.001) (See Fig. 4a). Beta diversity analysis based on the Non-metric Multidimensional Scaling (NMDS) method demonstrated distinct grouping of gut bacterial communities of Indian and Finnish children (Fig. 4b). Additionally, a total of 213 genera (63.6%) were found to be common amongst Indian and Finnish children, while 65 genera (19.4%) and 57 genera (17%) were unique in Finnish and Indian children respectively (Fig. 4c).

Our investigation on the prevalence (presence/absence) of different bacterial groups gave deeper insight into the gut bacterial community structure of Indian and Finnish children (Supplementary Table S4). It was observed that phyla such as Chloroflexi and Thermi were prevalent in almost all Indian children as compared to the Finnish population (Welch’s *t*-test, p = <0.001) (Supplementary Table S4a). Likewise, a comparison between Indian and Finnish gut bacterial community showed that genera such as *Acidaminococcus, Catenibacterium, Fusobacterium, Megasphaera, Mitsuokella*, and *Slackia* were significantly prevalent in Indian children (Welch’s *t*-test, p = <0.001). While, genera such as *Adlercreutzia, Anaerofustis*, and *Coprobacillus* were highly prevalent in Finnish (Welch’s *t*-test, p = <0.001) as compared to Indian children (Supplementary Table S4c). Together, these results suggested that there are substantial differences in gut microbial community structure of Indian and Finnish children.

### Influence of *FUT2* secretor status on gut microbial composition

**Figure 5:**
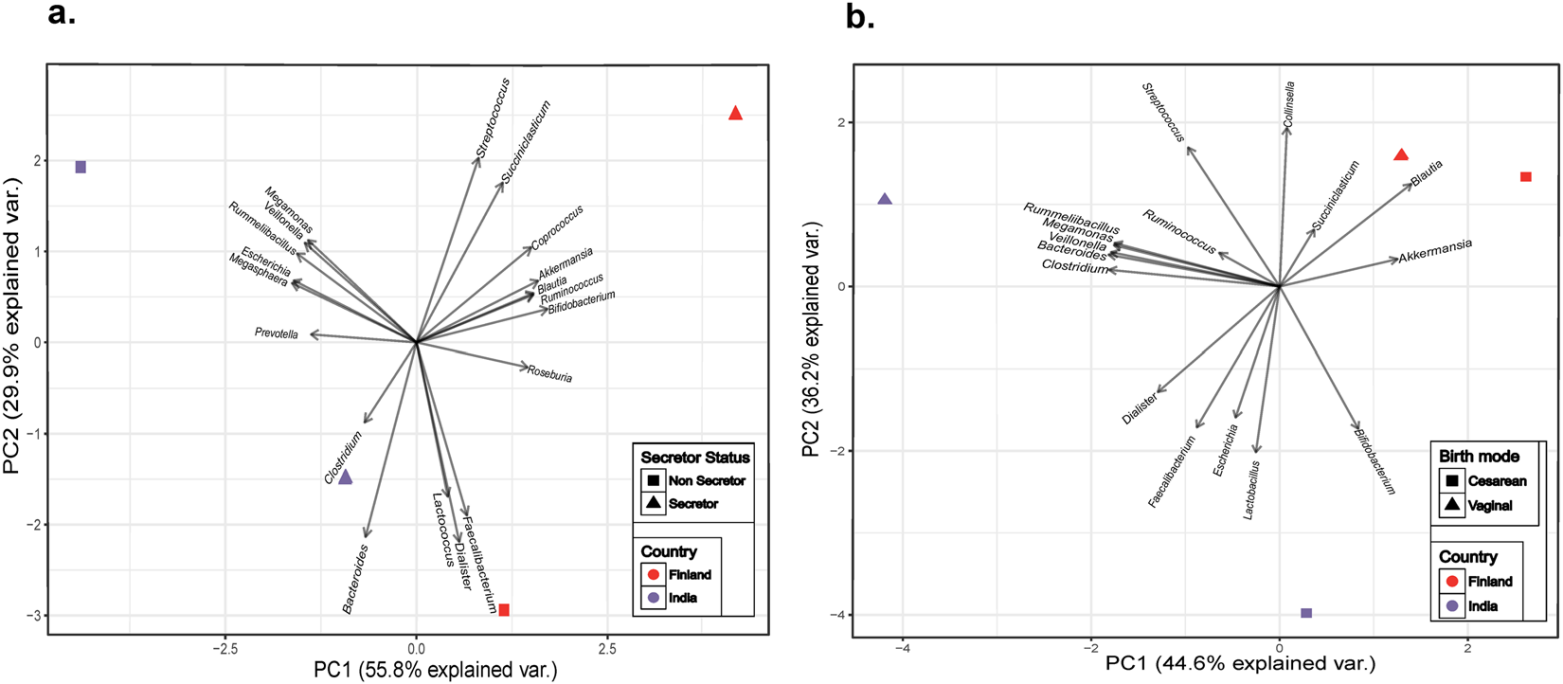
Principal Component Analysis. PCA was performed using the bacterial genera data; relative abundances were observed to be differing with *FUT2* secretor state and birth mode in both the populations (Supplementary Figs S4 and S5). Results were plotted according to the PC1 and PC2 scores with the percent variation explained by respective axis. Specific colors were used to indicate the region and different shapes to indicate either the secretor state (**a**) or birth mode (**b**). Arrows indicate bacterial genera with their names.

Our observations revealed that the gut microbiota composition is strongly associated with *FUT2* secretor status of an individual. Principal component analysis revealed that the influence pattern of *FUT2* variants differs substantially within the two populations (Fig. 5a), suggesting that different set of gut bacterial taxa are affected in a particular population as compared to the other. Phyla such as Actinobacteria and Verrucomicrobia were significantly higher in both Indian and Finnish secretor children (Welch’s *t*-test, q = <0.001), while Firmicutes and Proteobacteria were abundant in the non-secretor children in both the population (Welch’s *t*-test, q = <0.001) (Supplementary Fig. S3a and c). However, Bacteroidetes were found higher in Indian secretors (Welch’s *t*-test, q = <0.001), whereas they were observed to be higher in Finnish non-secretors (Welch’s *t*-test, q = <0.001).

The abundance of bacterial families such as *Bifidobacteriaceae, Ruminococcaceae, Verrucomicrobiaceae* and *Enterobacteriaceae* (Welch’s *t*-test, q = <0.001) exhibited a significant association with the secretor status of children in both populations (Supplementary Fig. S4a and c). *Bifidobacteriaceae* and *Verrucomicrobiaceae* were significantly abundant (Welch’s *t*-test, q = <0.001) in secretors as compared to non-secretors, while *Veillonellaceae* and *Enterobacteriaceae* were found at significantly higher levels (Welch’s *t*-test, q = <0.001) in non-secretors. Additionally, bacterial families like *Coriobacteriaceae, Prevotellaceae* and *Clostridiaceae* were highly abundant in secretors when compared to non-secretors of the Indian population (Welch’s *t*-test, q = <0.001), while non-secretors harbored higher *Planococcaceae* as compared to secretors (Welch’s *t*-test, q = <0.001) within Indian cohort. *Lachnospiraceae* group was highly significant in secretor children (Welch’s *t*-test, q = <0.001) within Finnish cohort (Supplementary Fig. S4a and c)

Further investigations to understand the effect of *FUT2* secretor status on different bacterial genera demonstrated that within the Finnish population, *Coproccus, Succiniclasticum, Streptococcus*, and *Blautia* were highly abundantin secretor children (Welch’s *t*-test, q = <0.001), while bacteria belonging to genus *Lactococcus* were highly abundant in non-secretors (Welch’s *t*-test, q = <0.001). Similarly, genera like *Megamonas, Veillonella, Rummeliibacillus*, and *Megasphaera* were abundant in Indian children with non-secretor status (Welch’s *t*-test, q = <0.001); However the genera *Prevotella, Roseburia* and *Clostridium* were significantly higher in secretors (Welch’s *t*-test, q = <0.001). Bacterial genera such as *Bifidobacterium, Akkermansia*, and *Ruminococcus* were observed to have higher abundance in secretors, whereas non-secretors of both Indian and Finnish cohort showed higher abundance of *Escherichia*. (Supplementary Fig. S4b and d). Collectively, this analysis indicated that *FUT2* secretor status did influence the gut microbial composition in both the cohorts; the pattern of which differed between these populations.

### Influence of Mode of delivery on gut microbial composition

Gut bacterial communities were also observed to have a significant association with the mode of delivery of an individual. We also performed principal component analysis to assess whether the influence of birth mode on the gut microbial composition of Indian children was similar to that of Finnish children. Our analysis showed substantial differences in the pattern, suggesting that different set of taxa are affected due to birth mode variants (cesarean VS. vaginal) between these populations (see Fig. 5b). Amongst the significantly affected bacterial phyla, Bacteroidetes and Firmicutes were higher (Welch’s *t*-test, q = <0.001) in vaginally born children in both Indian and Finnish population, while Proteobacteria and Verrucomicrobia were seen to be more abundant (Welch’s *t*-test, q = <0.001) in cesarean born children. It was noted that the phylum Actinobacteria was significantly abundant (Welch’s *t*-test, q = <0.001) in cesarean born as compared to vaginally born Finnish children (Supplementary Fig. S3b and d).

Furthermore, mode of delivery had a significant impact on bacterial families such as *Enterobacteriaceae, Verrucomicrobiaceae* (abundant in Cesarean born, Welch’s *t*-test, q = <0.001), *Bacteroidaceae, Veillonellaceae* and *Streptococcaceae* (abundant in vaginally born, Welch’s *t*-test, q = <0.001) in Finnish and Indian children as well (Supplementary Fig. S5a and c). Genera such as *Escherichia, Akkermansia*, *Bacteroides*, and *Streptococcus* had a similar impact of birth mode within both the cohorts under study. Former two being higher in cesarean born, while the others abundant in vaginally born children. Conversely, distinct influence on abundance of genera *Bifidobacterium* and *Faecalibacterium* were noted (Supplementary Fig. S5b and d) between these populations. Also, genera like *Dialister, Succiniclasticum, Ruminococcus*, and *Blautia* were abundant in vaginally born children as compared to cesarean within Finnish cohort (Welch’s *t*-test, q = <0.001). It was also noted that vaginally delivered Indian children harbored higher abundance of *Megamonas, Veillonella, Rummeliibacillus, Clostridium, Megasphaera*, and *Collinsella*, while cesarean born Indian children harbored higher *Lactobacillus* as compared to their respective counterparts (Welch’s *t*-test, q = <0.001). Together, these observations showed that birth mode had an influence on the gut microbial composition. However, the pattern differed when compared between Indian and Finnish population.

### Differences in the predicted functions of gut bacterial community

**Figure 6:**
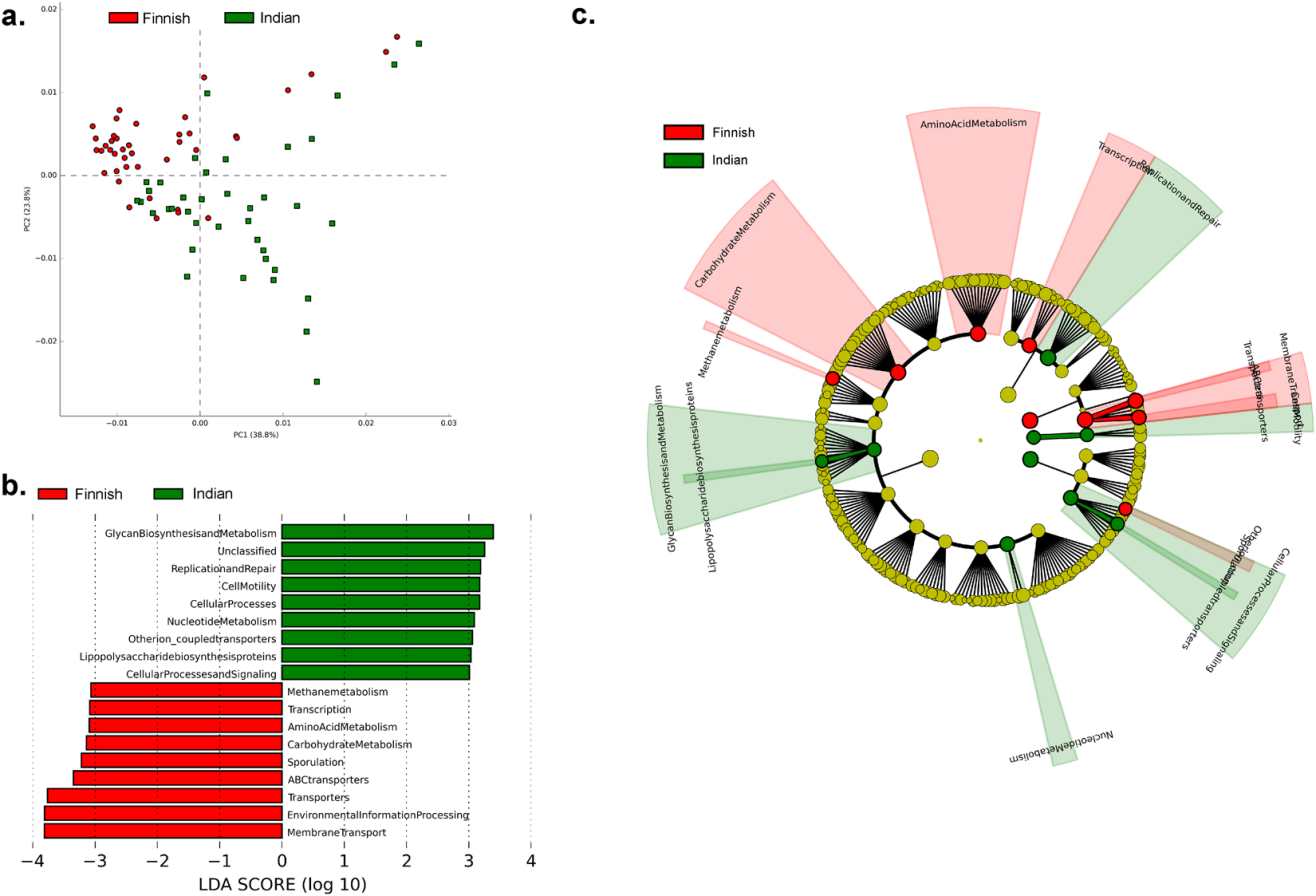
Imputed metagenomic functions of the gut bacterial community. (**a**) PCA plot is illustrating differences in metabolic functions of the gut bacterial community of Finnish (red) and Indian (green) children. (**b**) Bar chart is representing specific metabolic function differential abundant in Finnish and Indian children (Effect size filter = >2.0). (**c**) Cladogram representing differences in abundance of microbial genes between the gut bacterial community of Finnish and Indian children contributing in specific metabolic pathways.

We further analyzed the microbial community data to predict their metabolic functions. This analysis revealed significant differences between the functional profile of Indian and Finnish gut bacterial community as illustrated in Fig. 6. The PCoA plot (see Fig. 6a) represents distinct clustering of Finnish and Indian samples based on the predicted metabolic functions of the respective bacterial community. Also, the microbiota of Indian children was predicted to have significantly higher genes involved in glycan biosynthesis and metabolism, cell motility, lipopolysaccharide (LPS) biosynthesis proteins (Kruskal-Wallis test, Linear Discriminant analysis (LDA) score effect size filter = 2). Likewise, Finnish gut microbiota had significantly higher genes contributing to processes such as Carbohydrate metabolism, Methane metabolism, and transport systems (Kruskal-Wallis test, LDA score effect size filter = 2) as represented in Fig. 6b. Additional information of differentially abundant genes between Indian and Finnish population is illustrated in Supplementary Fig. S6.

**Figure 7:**
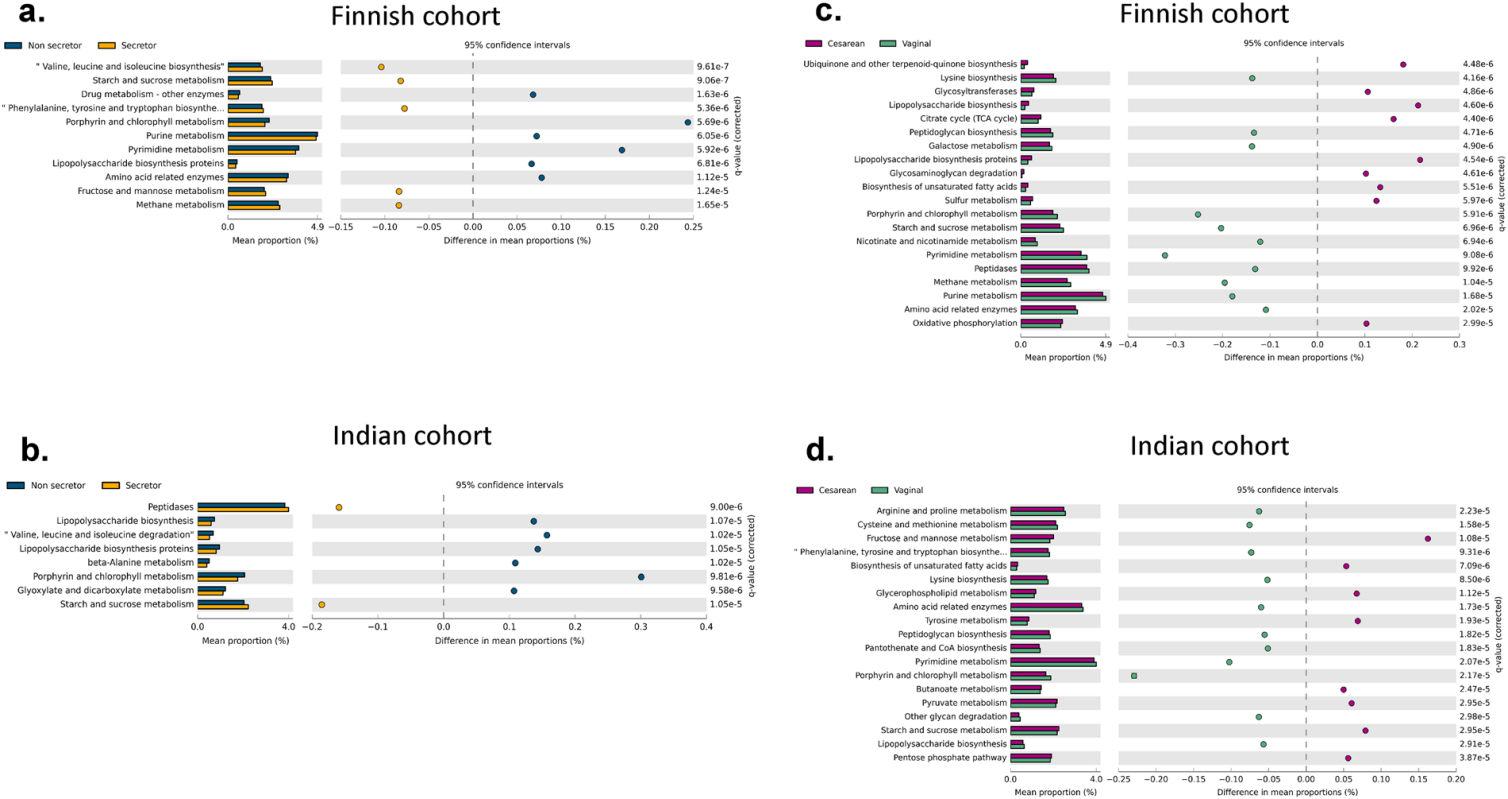
Effect of *FUT2* secretor status and birth mode on the pattern of microbial metabolic functions. (**a** and **b**) Extended bar plots are representing significantly different KEGG orthologs associated with microbial metabolic functions between secretor and non-secretor children of the Finnish and Indian population respectively. (**c** and **d**) Extended bar plots are representing significantly different KEGG orthologs associated with microbial metabolic functions between vaginally born and cesarean born children of the Finnish and Indian population respectively. Appropriate effect size filter was employed to represent highly affected features.

Furthermore, cladogram analysis based on KEGG orthologs using Linear discriminative analysis Effect Size (LEfSe) also supported the previous observation of distinct metabolic profiles of Indian and Finnish children (see Fig. 6c). We directed our investigation to decipher association of *FUT2* secretor status and birth mode with functional profiles of the bacterial community. This approach revealed significant association of secretor status and abundance of genes involved in starch and sucrose metabolism (higher in secretors, Welch’s *t*-test, q = <0.001), lipopolysaccharide biosynthesis proteins and porphyrin and chlorophyll metabolism (more abundant in non-secretors, Welch’s *t*-test, q = <0.001) amongst Indian and Finnish children (see Fig. 7a and b). Moreover, genes contributing to methane metabolism and synthesis of Branched Chain Amino Acids (BCAA): valine, leucine, and isoleucine were found to be higher in secretors as compared to the non-secretor status of Finnish cohort (Welch’s *t*-test, q = <0.001). Beta-Alanine metabolism and glyoxylate and dicarboxylate metabolism genes were found to be higher in non-secretors as compared to the secretor children of Indian cohort (Welch’s *t*-test, q = <0.001).

The impact of mode of delivery on the abundance of carbohydrate metabolism (fructose, mannose, sucrose and starch metabolism, etc.) genes differed between the two populations. Interestingly, these genes which were higher in vaginally born Finnish children, were observed to be abundant in cesarean born Indian children (see Fig. 7c and d). However, mode of delivery had a similar effect on the functional features of biosynthesis of unsaturated fatty acids (observed highly abundant in cesarean born children, Welch’s *t*-test, q = <0.001) in both the cohorts under study. Also, these factors had a significant influence on other metabolic features of bacterial community as illustrated in Fig. 7a–d. Altogether, these results indicate that the metabolic profiles of gut microbiota were substantially different amongst two populations and also between *FUT2* and birth mode variants within the population.

## Discussion

In this cross-sectional comparative study, we found that the gut microbial composition of Finnish and Indian children differed significantly, with a distinct set of bacterial taxa being predominant in each population. Our investigation further reveals the association of *FUT2* and birth mode variants with gut microbial composition at 13–14 years of age, patterns of which were discrete in the two geographically and ethnically distinct populations considered in this study.

We quantified a select group of bacterial species which represent the early colonizers of the gut using qPCR, and were previously reported to be associated with factors such as nutrition, allergy, and immune-modulation in the western population^44, 45^. We evaluated their prevalence in the gut microbiome at 13–14 years of age in both populations, which provided us primary leads about differences in the gut microbial community structure between the two populations. Interestingly, it was observed that few *Bifidobacterium* species were present in higher number in one population, while lower in another. These observations could be related to the differences in their genomes encoding for varied, complex polysaccharide degrading enzymes (CAZymes)^46–48^. Further, the abundance of *Akkermansia*, a known mucin degrader in the intestine, was also found to be substantially higher in Finnish children. This bacterium is shown to possess a strong negative correlation with high-fat diet^49–51^, indicating probable differences in the fat content consumed by these populations. Thus, these findings are indicative of the fact that such common intestinal colonizers are responsive to the distinct dietary habits across the globe, playing a key role in shaping the gut microbiome^7, 39^. We also performed principal component analysis, which indicated that out of the targeted bacteria in the qPCR experiment, only a few i.e. *Clostridium* species could explain the variation and distinct clustering of the Indian population. We further tried to correlate the qPCR data with that obtained from the 16S rRNA gene-based microbial profiling. Although not significant (except for *Bifidobacterium*), similar differential trend in the abundances of these genera was observed in the HTS data. This unsubstantial discrepancy could be attributed to the technical differences, such as the use of species-specific primers in qPCR, as against universal bacterial primers in the HTS. Collectively, this integrated analysis of qPCR and HTS data suggested an urge for exploring such untapped and functionally important taxa in the Indian population.

Our analysis based on the PCR-DGGE and HTS clearly demonstrated the extent of distinctness and diversity in the gut microbial composition of children of similar age but from distinct populations. The outcomes of our multi-pronged approach suggests that the gut microbial communities harbored by Finnish and Indian children are not only distinct but are driven by specific bacterial groups such as *Prevotella*, *Megasphaera* (Indian subjects), *Blautia* and *Bifidobacterium* (Finnish subjects). It also provided important information about the differential abundances of genera such as *Blautia* and *Ruminococcus*, increased numbers of which are reported to be induced by whole grain consumption^52^. Further, these differences were also reflected in the predicted functions of gut microbial community when compared between populations. Thus such variations in the microbial composition observed through qPCR and HTS, as well as in their imputed metabolic functions could be collectively attributed to the response of dietary and genetic variances between these two geographically distinct countries.

The key caveat that needs to be considered in this study is the sample size (especially for *FUT2* non-secretors) which doesn’t allow generalization about the entire population. However, this study gives us clues about differences within the same age groups and especially in growing children. This pilot study has generated a preliminary report which already shows promise regarding the impact that these factors have on the intestinal microbiota. Given the total populations of the two countries being considered in this “Pilot study” and the proportion of non-secretors globally (~30% with minor allele frequency A = 0.3217 globally; www.ncbi.nlm.nih.gov/variation/tools/1000genomes), extensive sampling would be needed for generalizing these findings at population level.

The sample size conundrum led us to further employ a statistically robust approach to determine whether similar influence patterns of host elements exist in two distinct populations. Our analysis revealed a strong association of gut microbial composition with secretor status of the subject amongst both the populations. According to previous studies, most of the taxa that were found higher in secretor subjects, are known to harbor enzymatic machinery like α-L-fucosidases and β-galactosidases which enable these bacterial groups to degrade and utilize the glycan chains of the blood group antigens expressed in the intestine^53, 54^. Besides, the exact reason for the higher abundance of genera such as *Streptococcus* (Finnish children) and *Clostridium* (Indian children) in secretor subjects is unclear but could be concomitant to their ability to use the human mucin glycans and compete for intestinal colonization^48, 54^. A key result of this study is the association of organisms other than LAB (as reported before) with the *FUT2* genotype. Interestingly, it was noted that the genes encoding for lipopolysaccharide (LPS) biosynthesis were significantly more abundant in non-secretors in both the populations. Previous studies suggest that high level of LPS production triggers the immune responses leading to systematic inflammation and dysbiosis in the gut^55^. These observations collectively indicate that *FUT2* polymorphism (non-secretor status) contributes to the selection of bacterial community and in turn modulates the innate immune responses in the gut, which makes an individual more prone to inflammation associated diseases like IBD, obesity, T2D^5, 23–25^. However, from our investigations, it was noted that the pattern of influence of *FUT2* variants on gut microbiota differed amongst the Indian and Finnish children. These differences in the influence could be related to the distinct dietary habits and environmental conditions which are known to play a key role in shaping the gut microbial composition, as also described by Kashyap *et al*.^56^. Despite the fact that mode of delivery is associated with specific gut bacterial groups in the neonates, very few studies have been directed towards understanding this association in the later life^30–32, 57^ and comparing those effects amongst differing geographical locations. Our efforts towards understanding this association in 13-14 years children (age of adolescence) revealed that there is a long-term effect of mode of delivery on the gut microbiome of an individual. Of particular note are higher abundances of Actinobacteria and Proteobacteria in cesarean born children. Similar patterns are reported in the patients suffering from gastrointestinal diseases such as IBD^58^, suggesting that cesarean born children may have increased risk of such diseases due to microbial alterations. As also for *FUT2* secretor state, differences were also reflected in the predicted functional profiles about the birth mode variants in both the populations. However, the pattern of influence differed substantially between the two populations as clearly evident from the principal component analysis. Our observations suggest that such variances in pattern indicate the role of gut microbial alterations, caused due to discrete environmental challenges faced by the mother and child during birth (delivery) in these two populations. Further, the nature of microbiota of the Indian subcontinent suggests more extensive studies being needed before concluding if factors such as secretor status and birth mode can partition the population in a meaningful way.

Sample size is an important caveat of this study which could have masked the differences occurring within low abundance taxa between groups. We, therefore, suggest that future comparisons of this type will require power analysis (for estimated sample size) taking into consideration factors investigated here. Despite these shortfalls, our preliminary study using such factors does reveal for the first time differences in gut microbial composition in these distinct populations. Factors such as age, socio-economic status, and ethnicity which are a substantial part of environmental variables remain largely consistent within each cohort thus reducing confounding effects on observed differences.

Altogether, we hypothesize that there exists a possibility that well-documented knowledge obtained about how exactly *FUT2* secretor state and birth mode influence the gut microbial community, may not be valid across different populations. Our findings emphasize the need for careful considerations of these differences during comparison and also in the view of designing any future studies, specifically those involving international intervention techniques aiming at remission of microbial alterations caused due to such host and environmental elements. It thus highlights the need for thorough understanding of the influence of such associations in a particular population with extensive sampling, especially in a heterogeneous community such as India.

In summary, we demonstrated that there are differences in the gut microbial community of 13–14-year-old children of the Indian and Finnish population. We also showed that there are differences in the mechanism by which host genotype and other elemental host factors such as birth mode influence the gut microbiome in distinct populations. Also, our findings underscore the importance of such comparisons in relation with these key determinants (*FUT2* and birth mode variants) involved in shaping the gut microbiome.

## Methods

### Sample collection

A total of 99 children (Indian, n = 47 and Finnish, n = 52), of age 13–14 years old, with no indications of infections during the sampling time and with a medication history of no antibiotic consumption for three months preceding sample collection, were recruited for the study. The study was approved by the ethics committees of National Centre for Cell Science, King Edward’s Memorial Hospital research Centre and Turku University Hospital. Written informed consent from the parents/guardians of the participants was obtained using the methods approved according to the guidelines of respective centers. All methods and experiments were performed by following relevant guidelines and regulations. A detailed questionnaire was designed and was addressed by the parents/guardians of the participants. The demographic and general characteristics of the cohorts under study are described in Table 1. The questionnaire included information about the medical and medication history etc. Fecal and EDTA anticoagulated whole blood samples were collected and stored at −80 °C until further processing.

### DNA extraction

Fecal and blood samples collected from the subjects were processed for total DNA extraction using QIAmp Mini stool DNA extraction and Blood & Tissue DNA extraction kit (Qiagen, USA) following the manufacturer’s instructions. Extracted DNA samples were quantified using NanoDrop spectrophotometer ND1000 (Thermo Scientific, USA).

### SNP analysis

Single Nucleotide Polymorphism (SNP) analysis to determine the *FUT2* SNP rs601338 was carried out by using the genotyping method. Briefly, the exon region (exon 2) of *FUT2* gene was amplified using specific primers as described previously^28^. Sequencing of the amplicon was carried out on ABI 3730 XL sequencer (Applied Biosystems, USA). Mutation analysis was carried out using SeqScape 2.6.0 (Applied Biosystems, USA) and heterozygous bases were detected (see details in Supplementary Table S5).

### DGGE based Bacterial community fingerprinting

DGGE of the DNA extracted from fecal samples was carried out as described in previous studies to obtain a bacterial community fingerprint^15^. PCR primers F357: 5′-GCCGCCCGCCGC

GCCCCGCGCCCGGCCCGCCGCCCCCGCCCCTACGGGAGGCAGCAG-3′ and R518: 5′-ATTACCGCGGCTGCTGG-3′ were used as described previously^15, 59^ to target the total bacterial community with a gradient range of 35–70%. The standard bacterial strains used are listed in Supplementary Table S2. The gel images were processed using Bionumerics tool version 6.6 (Applied Maths, Belgium) and were analyzed as described by Kumar *et al*., 2013^26^. Furthermore, a dendrogram was constructed for comparison of the total bacterial community between Indian and Finnish subjects as described by Endo *et al*.^15^. Euclidean distances based on the data obtained from Bionumerics were calculated to construct PCoA plots.

### Quantification of specific bacterial species

qPCR-based quantification of 12 bacterial groups from the fecal samples was carried out as described previously by Collado *et al*.^60^. List of bacterial species along with the primers used is presented in Supplementary Table S1.

### Library preparation and high throughput sequencing

DNA extracted from the fecal samples was used to amplify the V4 region of 16 S rRNA gene to determine the gut bacterial community structure. Primer set 515 F/806 R was employed to target the V4 region as described in earlier studies^61, 62^. The amplified products were further subjected to library preparation and sequencing on the Illumina HiSeq. 2000 platform as per the manufacturer’s instructions (Illumina technologies, USA). Sequencing was performed using V4 paired-end (2 × 250 bp) chemistry. The paired end reads obtained, were quality checked using the FastQC tool [Andrews S. (2010). FastQC: a quality control tool for high throughput sequence data. Available online at http://www.bioinformatics.babraham.ac.uk/projects/fastqc] and assembled using FLASH assembler tool^63^. Details of the sequencing are added in Supplementary Table S5.

### Community structure analysis

The assembled reads were used for community structure analysis using QIIME pipeline^64^. OTU picking method was carried out using UCLUST closed reference method^65^, and the representative OTUs were assigned taxonomy using RDP classifier method^66^ with Greenegenes (13_8 release) as reference dataset^67^. Alpha and beta diversity analysis were performed, and further statistical analysis was carried out using R and STAMP^68^. Normalization of the data was carried out using rarefaction step included in QIIME package. A detailed description of the normalization carried out for various comparisons is given in the statistical methods and analyses section.

### Prediction of metabolic functions of the bacterial community

Metagenomic prediction tool ‘Phylogenetic Investigation of Communities by Reconstruction of Unobserved States’ (PICRUSt)^69^ was utilized to determine the metabolic functions of the bacterial community. Nearest Sequence Taxonomic Identity (NSTI) values were obtained for each sample and samples with NSTI values more than 0.10 were excluded from further analysis to avoid false predictions as described by Langille *et al*. 2013 (which suggest the NSTI values for human samples to be lesser than 0.10 with higher prediction accuracy)^69^. Metagenomic predictions were obtained based on KEGG Orthology (KO) and were further analyzed using LEfSe^70^ to obtained differentially abundant genes, especially those involved in various metabolic processes of the bacterial communities. This approach was used to carry out comparisons of imputed metabolic functions of the bacterial community, between the Finnish and Indian population and also to decipher effect of *FUT2* secretor status and birth mode on metabolic functions within these populations.

### Statistical methods and analyses

UPGMA based dendrogram analysis using Pearson similarity coefficient was performed on the PCR-DGGE fingerprinting data, while UPGMA based dendrogram analysis was carried out using STAMP for qPCR data. Euclidean distance based matrices were used to construct PCoA plots for DGGE data using QIIME. Statistical packages provided in the STAMP tool were utilized to determine the differences in the bacterial community as well as the imputed metabolic functions within groups under consideration. To overcome the sample size bias during the comparisons carried out between secretor vs. non-secretor and vaginally born vs. cesarean born children for both the populations, a randomization approach was used. This approach included consolidating the OTU data of all the samples from a particular group (e.g. secretor or non-secretor) thus considering the total obtained set as one microbial community representing a single group. Furthermore, normalization was carried out against the other respective group using three iterations of randomization (triplicate), and data for all the replicates obtained was further used for comparison. Welch t-test (two-sided) with Benjamini-Hochberg FDR correction (q-value filter = 0.05 and appropriate effect size filter) was applied for multiple comparisons considering the unequal variances of the study groups. Bar graph analysis was carried out using the non-parametric Mann-Whitney test in GraphPad prism for evaluating significant differences in abundances of specific genera between two groups. Furthermore, NMDS plots based on Bray-Curtis distance matrix were constructed to carry out beta diversity analysis. Factorial Kruskal-Wallis test (α = 0.05) was employed for inter-class comparisons with appropriate logarithmic LDA score filter using LEfSe.

### Data availability

The sequence data is made available at NCBI SRA submission with accession number SRP077882 (Bioproject ID PRJNA327770).

## Electronic supplementary material

Supplementary material
