# Supplementary material for: A cross-sectional comparative study of gut bacterial community of Indian and Finnish children
Source: Sci Rep. 2017 Sep 5;7:10555. doi: 10.1038/s41598-017-11215-y (PMC5585376; doi:10.1038/s41598-017-11215-y)
Supplement: Supplementary file 1 — Supplementary material [file 41598_2017_11215_MOESM1_ESM.pdf]

A cross-sectional comparative study of gut bacterial community of Indian and Finnish children

Shreyas V. Kumbhare[1,2†], Himanshu Kumar[3†], Somak P. Chowdhury[2,4], Dhiraj P. Dhotre[2], Akihito Endo[5], Jaana Mättö[6], Arthur C. Ouwenhand[7], Samuli Rautava[3,8], Ruchi Joshi[9], Nitinkumar P. Patil[10], Ravindra H. Patil[1], Erika Isolauri[8], Ashish R. Bavdekar[9], Seppo Salminen*[3], Yogesh S. Shouche*[2]

Affiliations:

[1]Department of Microbiology, R.C. Patel Arts, Science, and Commerce College, Shirpur, Dist. Dhule, Maharashtra, India, 425405.

[2]National Centre for Cell Science, Savitribai Phule University of Pune campus, Ganeshkhind, Pune, Maharashtra, India, 411007.

[3]Functional Foods Forum, Faculty of medicine, University of Turku, Turku, Finland, 20520.

[4] Max Planck Institute for Biogeochemisty, Jena, Germany - 07747

[5]Department of Food and Cosmetic Science, Tokyo University of Agriculture, Hokkaido, Japan.

[6]Finnish Red Cross Blood Service, Helsinki, Kivihaantie 7, Finland, 00310.

[7]Active Nutrition, DuPont Nutrition & Health, Kantvik, Finland, 02460.

[8]Department of Paediatrics, University of Turku, Turku, Finland, 20520.

[9]King Edward Memorial hospital research centre, Pune, Maharashtra, India 411011.

[10]Department of Microbiology, Smt. Chandibai Himathmal Mansukhani College, Ulhasnagar, Thane, Maharashtra, India, 421003.

*Correspondence and requests for materials should be addressed to Y.S.S. (email: yogesh@nccs.res.in) or S.S. (email: sepsal@utu.fi)

Supplementary material:

Figures: 6 (Figure S1-S6)

Tables: 5 (Table S1-S5)

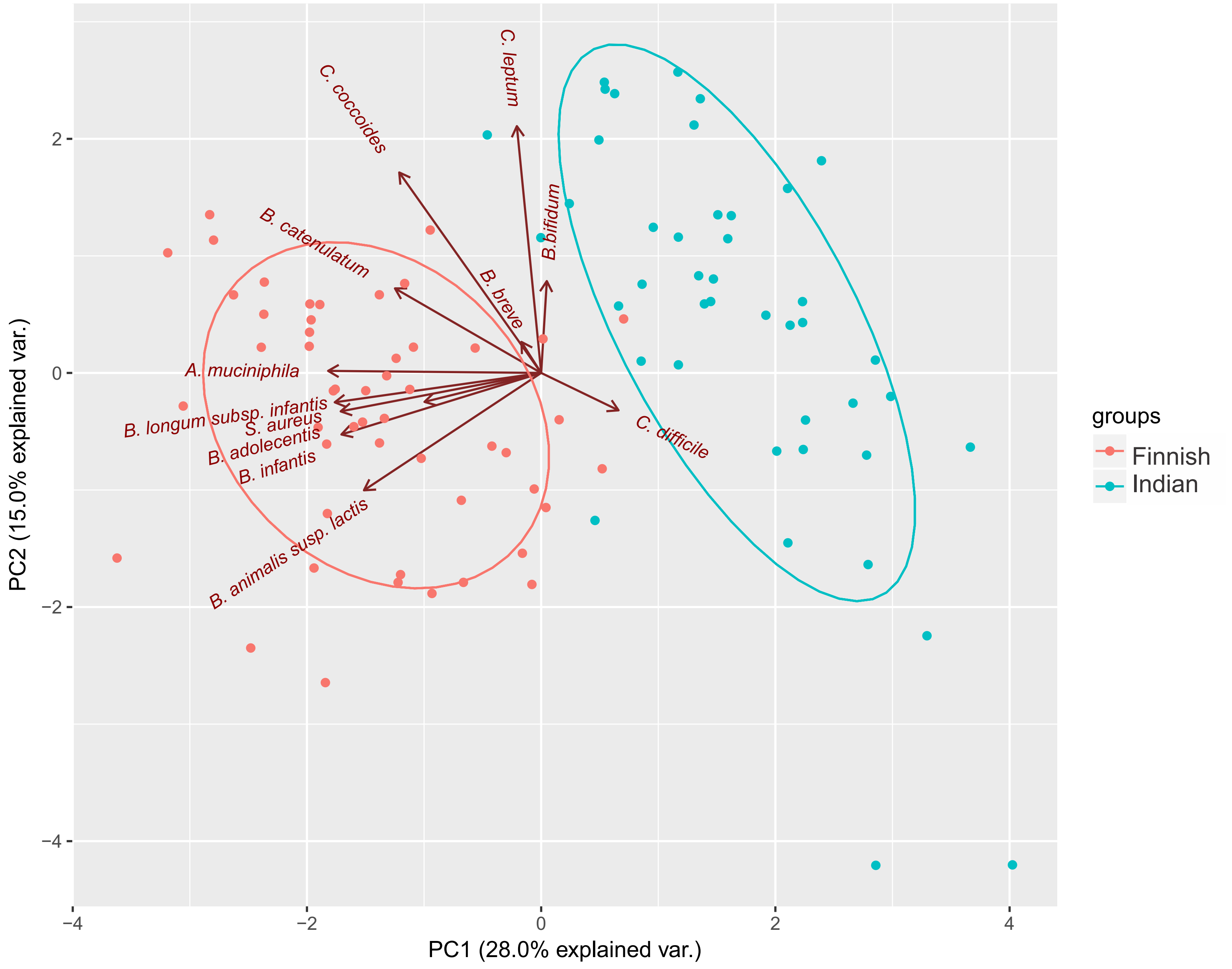

Supplementary Figure S1. Principal Component Analysis (PCA) illustrating the differences in abundances of different bacterial taxa and their contribution as components to explain the variation amongst Finnish (Red) and Indian children (Blue).

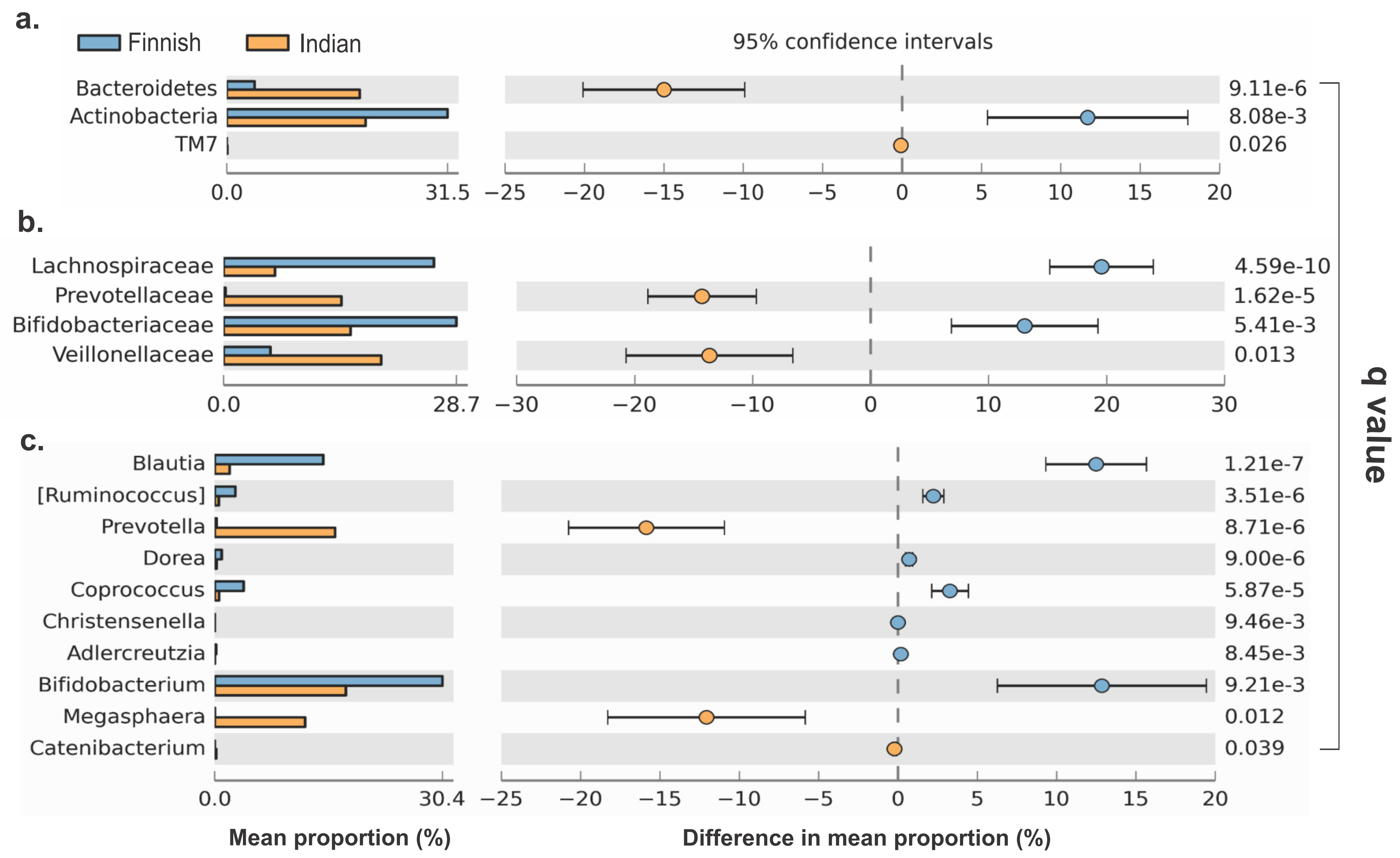

Supplementary Figure S2. Differences in bacterial composition between Indian and Finnish children (a) Extended bar plot exhibiting the differential abundances of bacterial phyla between Finnish (n=41) and Indian (n=42) children. **(b)** Extended bar plot exhibiting the differential abundances of bacterial families between Finnish (n=41) and Indian (n=42) children. **(c)** Extended bar plot exhibiting the differential abundances of bacterial genera between Finnish (n=41) and Indian (n=42) children.

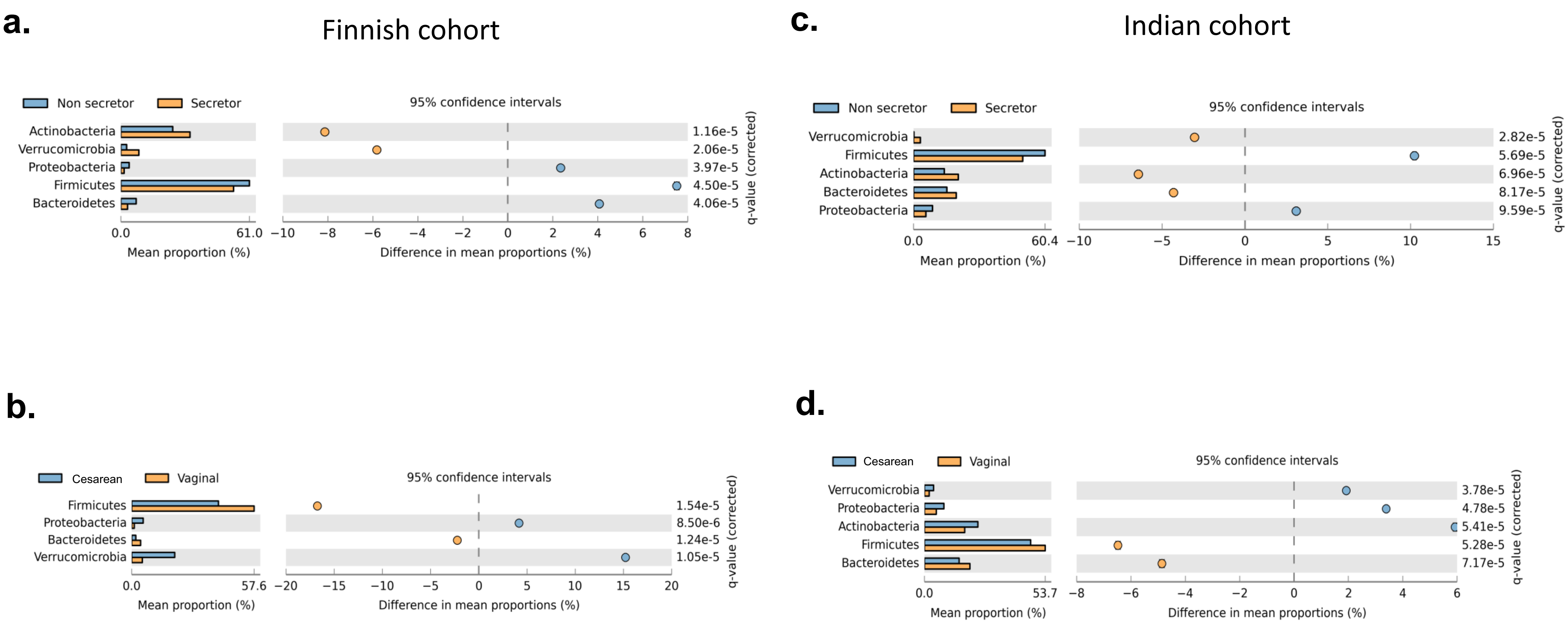

Supplementary Figure S3. Influence of *FUT2* secretor status and birth mode on various bacterial phyla (a & b) Extended bar plots representing significantly different bacterial groups at Phylum level within Finnish population (c & d) Extended bar plots representing significantly different bacterial groups at Phylum level within Indian population . Appropriate effect size filter was employed to represent highly affected groups.

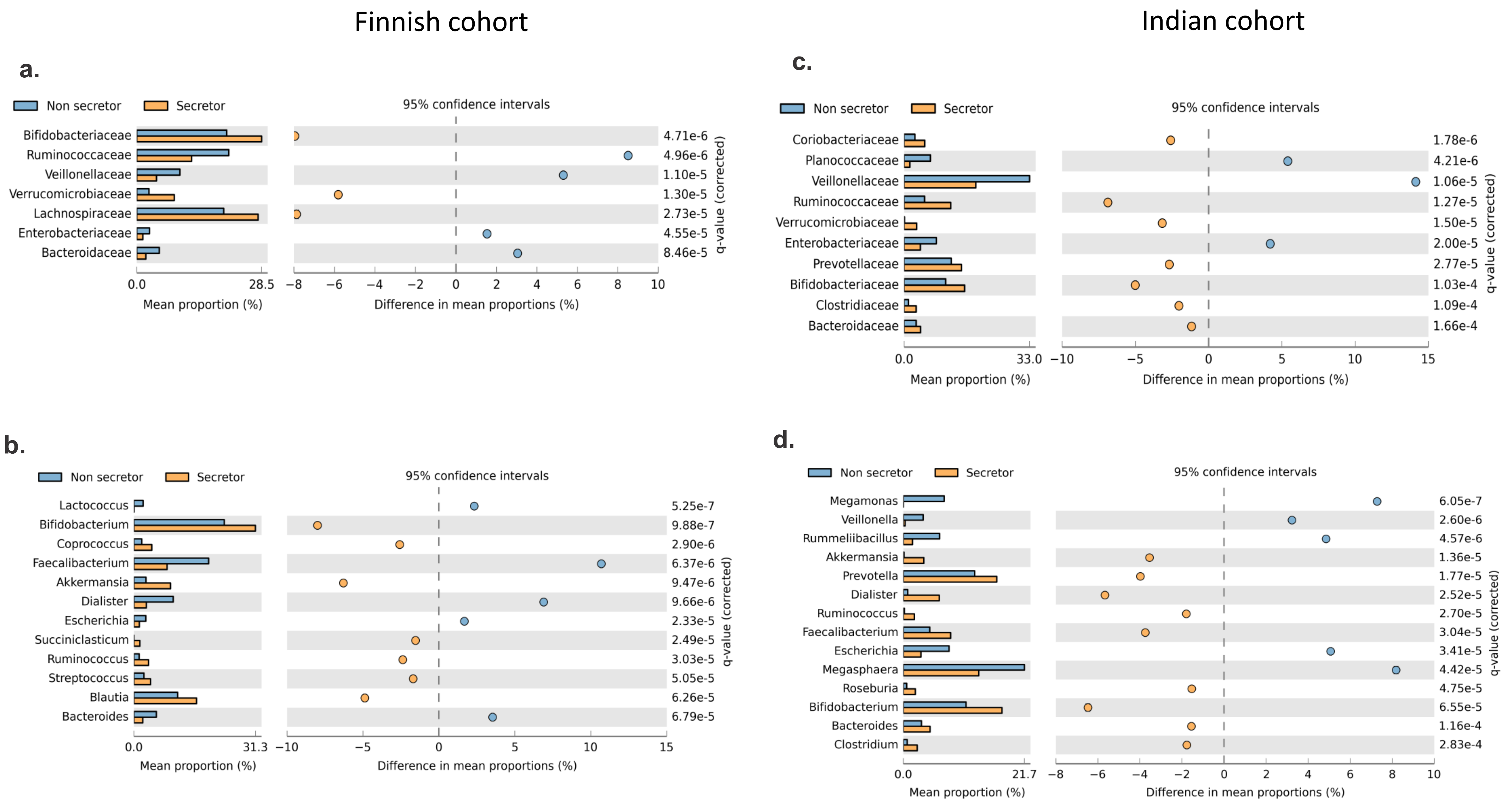

Supplementary Figure S4. Effect of FUT2 secretor status on gut bacterial composition (a & b) Extended bar plots representing significantly different bacterial groups at Family and Genus level respectively, between Finnish Secretor and Non-secretor children. **(c & d)** Extended bar plots representing significantly different bacterial groups at Family and Genus level respectively, between Indian Secretor and Non-secretor children. Effect size filter (>1.00%) was applied on the difference in mean proportions to obtain bacterial groups which are affected most amongst the total significantly different groups between Secretor and Non-secretor children.

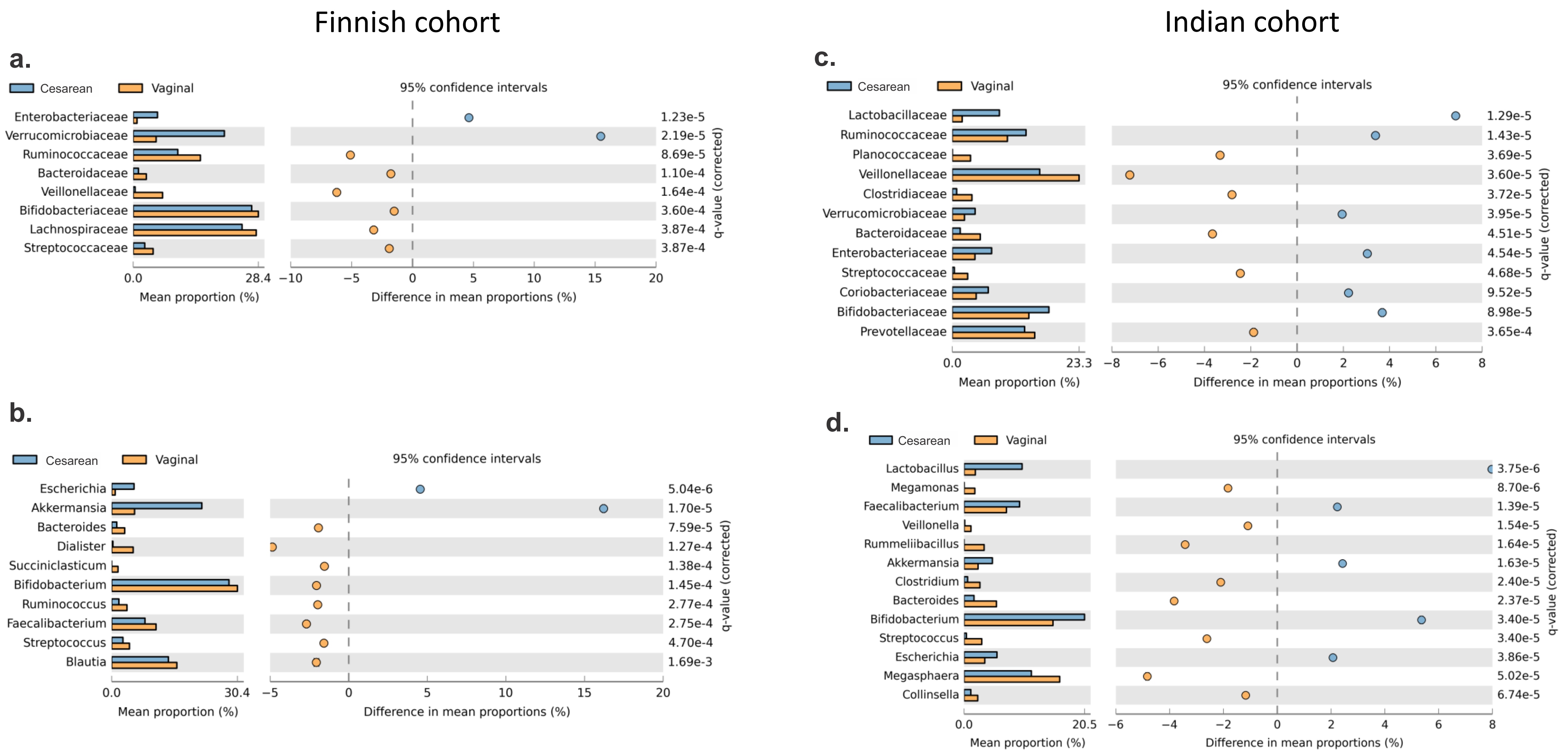

Supplementary Figure S5. Effect of birth mode on gut bacterial composition (a & b) Extended bar plots representing significantly different bacterial groups at Family and Genus level respectively, between Finnish Vaginally born and Cesarean born children. **(c & d)** Extended bar plots representing significantly different bacterial groups at Family and Genus level respectively, between Indian Vaginally born and Cesarean born children. Effect size filter (>1.00%) was applied on the difference in mean proportions to obtain bacterial groups which are affected most amongst the total significantly different groups between children with Vaginal and Cesarean birth mode.

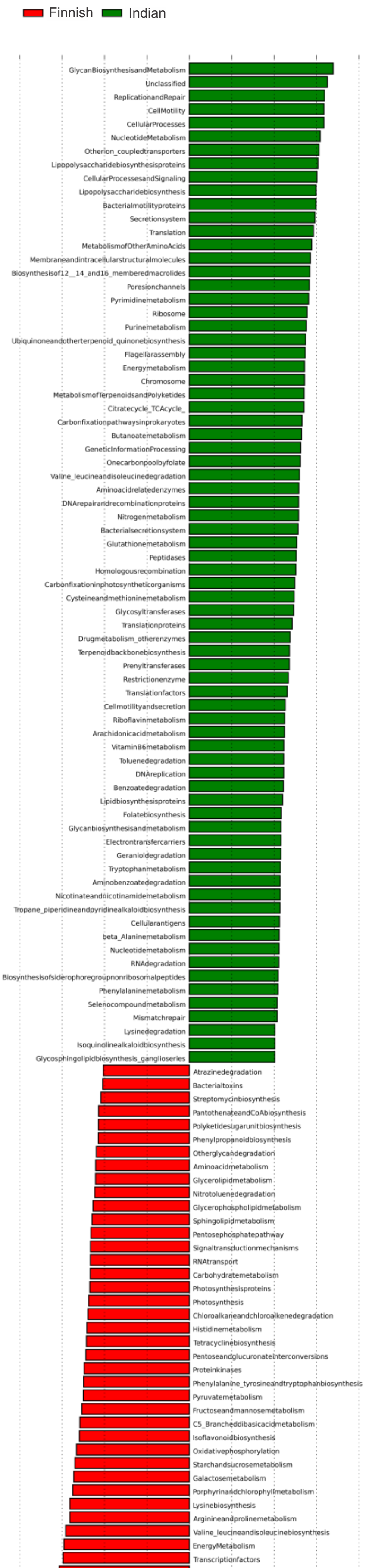

Supplementary Figure S6. Differences in imputed metabolic functions. The figure illustrates differential abundance of metabolic functions of the gut bacterial community analyzed using LEfSe based on the KEGG orthologs obtained from PICRUSt.

Target organism	Primers	Sequence from 5′ to 3′	Product length (bp)	Reference
Bifidobacterium genus	Bifido5'	GATTCTGGCTCAGGATGAACGC	552	Gueimonde et al. 2004
	Bifido3'	CTGATAGGACGCGACCCCAT		
Bifidobacterium adolescentis	Ado-Ang5'	GGATCGGCTGGAGCTTGCTCCG	279	Rinne et al. 2005
	Ado3'	CCCCGAAGGCTTGCTCCCAGT		
Bifidobacterium bifidum	Bifidum5'	TGACCGACCTGCCCCATGCT	278	Rinne et al. 2005
	Bifidum3'	CCCATCCCACGCCGATAGAAT		
Bifidobacterium breve	Breve5'	AATGCCGGATGCTCCATCACAC	288	Rinne et al. 2005
	Breve3'	GCCTTGCTCCCTAACAAAAGAGG		
Bifidobacterium catenulatum group	Caten5'	GCCGGATGCTCCGACTCCT	285	Rinne et al. 2005
	Caten3'	ACCCGAAGGCTTGCTCCCGAT		
Bifidobacterium longum subsp. infantis	BiINF-1	TTCCAGTTGATCGCATGGTC	828	Matsuki et al. 1999
	BiINF-2	GGAAACCCCATCTCTGGGAT		
Bifidobacterium animalis subsp. lactis	Lactis5'	ACCAACCTGCCCTGTGCACCG	194	Rinne et al. 2005
	Lactis3'	CCATCACCCCGCCAACAAGCT		
Bifidobacterium longum group	Longum5'	TTCCAGTTGATCGCATGGTCTTCT	831	Rinne et al. 2005
	Longum3'	GGCTACCCGTCGAAGCCACG		
Clostridium coccoides group	g-Ccoc-F	AAATGACGGTACCTGACTAA	440	Matsuki et al. 2002
	g-Ccoc-R	CTTTGAGTTTCATTCTTGCGAA		
Clostridium difficile	Cdif-F	TTGAGCGATTTACTTCGGTAAAGA	89	Penders et al. 2005
	Cdif-R	TGTACTGGCTCACCTTTGATATTYA		
Clostridium leptum subgroup	sg-Clept-F	GCACAAGCAGTGGAGT	239	Matsuki et al. 2004
	sg-Clept-R3	CTTCCTCCGTTTTGTACC		
Clostridium perfringens	CPerf165F	CGCATAAYGTTGAAAGATGG	105	Wise & Siragusa 2005
	CPerf269R	CCTTGGTAGGCCGTTACCC		
Staphylococcus aureus nuc gene	NUC1	GCGATTGATGGTGATACGGTT	270	Brakstad et al. 1992
	NUC2	AGCCAAGCCTTGACGAACTAA AGC		
Akkremansia muciniphila	S-St-Muc-1129-a-a-20 (AM1)	CAGCACGTGAAGGTGGGGAC	308	Collado et al. 2007
	S-St-Muc-1437-a-A-20 (AM2)	CCTTGCGGTTGGCTTCAGAT		

Supplementary Table S1. Quantification of bacterial groups. The table above describes list of bacteria that were quantified using respective primer sets with qPCR method.

Sr. No.	Bacterial strains used
1.	*Bacteroides fragilis* DSM 2151
2.	*Lactobacillus gasseri* DSM 20243
3.	*Leuconostoc mesenteroides subsp. mesenteroides* DSM 20343
4.	*Lactobacillus salivarius* DSM 20555
5.	*Bifidobacterium breve* DSM 20213
6.	*Bifidobacterium longum* DSM 20219
7.	*Bifidobacterium bifidum* DSM 20456
8.	*Bifidobacterium angulatum* JCM 7096

Supplementary Table S2. List of Bacterial strains used as reference standards for total bacterial diversity analysis using DGGE. Total genomic DNA from the mentioned strains was isolated and used further for PCR-DGGE analysis.

Comparison	Cohort	Chao1 (Mean ± SD)	Observed species (Mean ± SD)	Shannon (Mean ± SD)	Simpson (Mean ± SD)	Simpson reciprocal (Mean ± SD)
1. Country	Finnish (n = 41)	2599± 582.5	1792± 414.4	6.014± 0.9485	0.9297± 0.05	21.9± 14.73
	Indian (n = 42)	2300± 604.5	1499± 449	5.481± 0.8319	0.9164± 0.05	16.28± 8.577
	P-value	0.0674	0.0059	0.0135	0.1	0.1
2. Secretor status*	FS	7997± 165.1	6056± 14.15	7.261± 0.001	0.9742± 3.3E-05	38.77± 0.05
	FNS	7741± 0.0	5925± 0.0	7.574± 0.0	0.9796± 0.0	49.12± 0.0
	P-value	0.1	0.1	0.1	0.1	0.1
	IS	9025± 66.38	6779± 25.74	7.554± 0.001	0.9827± 8.54E-06	57.8± 0.02
	INS	7783± 0.0	5739± 0.0	6.835± 0.0	0.9728± 0.0	36.79± 0.0
	P-value	0.1	0.1	0.1	0.1	0.1
3. Birth Mode*	FV	8636± 179.6	6503± 23.12	7.46± 0.004	0.9768± 3.49E-05	43.14± 0.06
	FC	6039± 0.0	4824± 0.0	6.629± 0.0	0.9585± 0.0	24.11± 0.0
	P-value	0.1	0.1	0.1	0.1	0.1
	IV	10094± 182.2	7771± 22.59	7.493± 0.001	0.9814± 1.39E-05	53.76± 0.04
	IC	8179± 0.0	6511± 0.0	7.273± 0.0	0.9812± 0.0	53.25± 0.0
	P-value	0.1	0.1	0.1	0.1	0.1

Supplementary Table S3. Alpha diversity analysis. The table describes comparison of bacterial community within different study groups using various alpha diversity indices. * mark indicates that the analysis was carried out on the rarified consolidated data for comparison between secretor status and birth mode groups within Indian and Finnish population. P- value was calculated using Mann-Whitney U-test. Abbreviations: FS-Finnish Secretors, FNS- Finnish Non-Secretors, IS-Indian Secretors, INS- Indian Non-Secretors, FV- Finnish Vaginal, FC- Finnish Cesarian, IV- Indian Vaginal, IC- Indian Cesarian.

a.

Phylum	Indian	Finnish	P-value
Acidobacteria	69.05%	46.34%	0.03675928
Chloroflexi	92.86%	68.29%	0.00474412
Deferribacteres	16.67%	2.44%	0.02816974
Fusobacteria	88.10%	46.34%	3.1612E-05
Lentisphaerae	28.57%	7.32%	0.01144027
Thermi	97.62%	68.29%	0.00041543

b.

Family	Indian	Finnish	P-Value
Aerococcaceae	80.95%	39.02%	5.85191E-05
Alcaligenaceae	97.62%	82.93%	0.025897635
Alcanivoracaceae	47.62%	24.39%	0.027455198
Bacillaceae	73.81%	34.15%	0.000197988
Chitinophagaceae	59.52%	21.95%	0.000360808
Comamonadaceae	83.33%	51.22%	0.001625269
Deferribacteraceae	16.67%	2.44%	0.028169735
Dietziaceae	0.00%	9.76%	0.044025001
Eubacteriaceae	14.29%	78.05%	9.46953E-11
F16	47.62%	9.76%	9.26366E-05
Fusobacteriaceae	85.71%	34.15%	4.15408E-07
Gemellaceae	88.10%	68.29%	0.029747723
Geobacteraceae	9.52%	0.00%	0.044064044
Idiomarinaceae	35.71%	4.88%	0.000413541
Leptotrichiaceae	40.48%	17.07%	0.018263959
Leuconostocaceae	78.57%	56.10%	0.029458408
Micrococcaceae	95.24%	73.17%	0.006141834
mitochondria	80.95%	43.90%	0.000378757
Moraxellaceae	100.00%	82.93%	0.006533611
Neisseriaceae	100.00%	63.41%	2.20749E-05
Nocardiaceae	42.86%	19.51%	0.021497415
Oxalobacteraceae	83.33%	53.66%	0.003386777
Paraprevotellaceae	95.24%	58.54%	6.41572E-05
Pasteurellaceae	100.00%	90.24%	0.044025001
Peptostreptococcaceae	76.19%	92.68%	0.038691969
Planctomycetaceae	33.33%	9.76%	0.008687756
Planococcaceae	78.57%	39.02%	0.000173953
Pseudomonadaceae	80.95%	48.78%	0.001911265
Rhizobiaceae	38.10%	9.76%	0.002231553
Rhodobacteraceae	73.81%	41.46%	0.002558446
Rs-045	47.62%	4.88%	5.532E-06
Saprospiraceae	9.52%	0.00%	0.044064044
Shewanellaceae	92.86%	70.73%	0.009275119
Sphingomonadaceae	45.24%	14.63%	0.002044714
Succinivibrionaceae	85.71%	36.59%	1.49247E-06
Thermaceae	97.62%	68.29%	0.000415427
Victivallaceae	28.57%	7.32%	0.011440269
Xanthomonadaceae	95.24%	73.17%	0.006141834

Supplementary Table S4. Prevalence analysis. The charts depict prevalence of different bacterial groups within two cohorts. P-values mentioned in the chart indicate significantly different prevalence patterns of the bacterial community between Indian (n=42) and Finnish (n=41) subjects **(a)** Prevalence chart of bacterial community at Phylum level **(b)** Prevalence chart of bacterial community at Family level **(c)** Prevalence chart of bacterial community at Genus level.

c.

Genus	Indian	Finnish	P-Value
Abiotrophia	69.05%	24.39%	2.20651E-05
Acidaminococcus	50.00%	0.00%	1.15533E-07
Acinetobacter	100.00%	70.73%	0.00021629
Actinobacillus	45.24%	14.63%	0.002044714
Adlercreutzia	61.90%	100.00%	1.0415E-05
Alcanivorax	47.62%	24.39%	0.027455198
Alloiococcus	33.33%	14.63%	0.046582275
Alloscardovia	0.00%	12.20%	0.02340471
Amaricoccus	19.05%	0.00%	0.003435296
Anaerofustis	7.14%	70.73%	1.13298E-10
Bacillus	61.90%	31.71%	0.005420609
Butyricimonas	78.57%	56.10%	0.029458408
Candidatus Rhodoluna	0.00%	12.20%	0.02340471
Catenibacterium	78.57%	9.76%	6.65051E-13
Christensenella	21.43%	65.85%	2.28035E-05
Citrobacter	66.67%	31.71%	0.001195446
Coprobacillus	11.90%	58.54%	3.86312E-06
Eggerthella	64.29%	90.24%	0.004480762
Enterococcus	78.57%	56.10%	0.029458408
Epulopiscium	2.38%	14.63%	0.048655328
Flavisolibacter	42.86%	19.51%	0.021497415
Fusobacterium	80.95%	21.95%	4.42471E-09
Geobacillus	16.67%	2.44%	0.028169735
Geobacter	9.52%	0.00%	0.044064044
Holdemania	35.71%	68.29%	0.002626934
Kaistobacter	14.29%	0.00%	0.012457479
Kocuria	47.62%	19.51%	0.006276573
Lactococcus	61.90%	90.24%	0.002231553
Lautropia	26.19%	4.88%	0.007241058
Leptotrichia	38.10%	14.63%	0.014975593
Luteimonas	21.43%	2.44%	0.007738408
Megamonas	100.00%	70.73%	0.00021629
Megasphaera	100.00%	53.66%	7.02607E-07
Meiothermus	97.62%	60.98%	3.84327E-05
Micrococcus	52.38%	29.27%	0.032313462
Mitsuokella	71.43%	9.76%	3.5965E-10
Mucispirillum	16.67%	2.44%	0.028169735
Neisseria	97.62%	63.41%	8.75103E-05
Oribacterium	42.86%	12.20%	0.001526647
Pantoea	28.57%	7.32%	0.011440269
Paracoccus	57.14%	29.27%	0.009950177
Phascolarctobacterium	100.00%	90.24%	0.044025001
Planctomyces	33.33%	9.76%	0.008687756
Porphyromonas	88.10%	58.54%	0.002190427
Pseudoxanthomonas	52.38%	12.20%	5.48243E-05
Psychrobacter	57.14%	24.39%	0.002072842
Rhodanobacter	90.48%	65.85%	0.006652075
Rhodococcus	40.48%	19.51%	0.037400551
Rothia	92.86%	65.85%	0.002352529
Rummeliibacillus	59.52%	36.59%	0.03682443
Sarcina	45.24%	2.44%	3.22834E-06
Scardovia	85.71%	39.02%	4.87538E-06
Serratia	35.71%	14.63%	0.026899275
Shewanella	92.86%	70.73%	0.009275119
Shuttleworthia	26.19%	0.00%	0.00045201
Slackia	80.95%	29.27%	5.21671E-07
Sphingomonas	30.95%	7.32%	0.005955728
Succinatimonas	54.76%	24.39%	0.004258777
Succinivibrio	61.90%	14.63%	3.41718E-06
Sutterella	97.62%	80.49%	0.013599178
vadinCA11	11.90%	0.00%	0.023454786

#SampleID	Country	Sex	Birth mode	Genotype	Description	No. of assembled sequences (qvalue> 30)
f104	Finnish	Male	Vaginal	GG	Secretor	254448
f105	Finnish	Male	Vaginal	AA	Non secretor	209688
f109	Finnish	Female	Vaginal	AA	Non secretor	331404
f117	Finnish	Female	Vaginal	AA	Non secretor	287106
f118	Finnish	Female	Vaginal	GA	Secretor	234702
f121	Finnish	Male	Vaginal	GG	Secretor	284218
f123	Finnish	Female	Vaginal	GG	Secretor	251257
f125	Finnish	Female	Vaginal	GA	Secretor	253640
f127	Finnish	Female	Vaginal	GA	Secretor	233680
f129	Finnish	Male	C section	GG	Secretor	249334
f134	Finnish	Male	C section	GG	Secretor	296291
f138	Finnish	Female	Vaginal	AA	Non secretor	344038
f141	Finnish	Male	Vaginal	GG	Secretor	333455
f143	Finnish	Female	Vaginal	GG	Secretor	211524
f146	Finnish	Female	C section	GA	Secretor	299514
f149	Finnish	Male	Vaginal	GA	Secretor	217066
f154	Finnish	Male	Vaginal	GG	Secretor	354177
f155	Finnish	Male	Vaginal	GA	Secretor	227492
f158	Finnish	Male	Vaginal	GA	Secretor	195611
f161	Finnish	Female	Vaginal	GA	Secretor	184752
f162	Finnish	Male	C section	GG	Secretor	332247
f163	Finnish	Male	Vaginal	AA	Non secretor	328651
f164	Finnish	Female	Vaginal	GG	Secretor	296484

f172	Finnish	Female	Vaginal	GG	Secretor	265710
f176	Finnish	Female	C section	GG	Secretor	239938
f181	Finnish	Male	Vaginal	GA	Secretor	200341
f182	Finnish	Male	C section	GG	Secretor	200995
f183	Finnish	Male	Vaginal	GA	Secretor	261592
f198	Finnish	Male	Vaginal	GG	Secretor	220988
f200	Finnish	Male	Vaginal	GG	Secretor	274442
f202	Finnish	Male	Vaginal	AA	Non secretor	223052
f207	Finnish	Female	Vaginal	GA	Secretor	468711
f209	Finnish	Female	Vaginal	GG	Secretor	538509
f212	Finnish	Female	Vaginal	GG	Secretor	398343
f218	Finnish	Male	Vaginal	GA	Secretor	310761
f227	Finnish	Female	Vaginal	GA	Secretor	448268
f228	Finnish	Female	Vaginal	GA	Secretor	519704
f231	Finnish	Female	Vaginal	AA	Non secretor	419947
f238	Finnish	Male	Vaginal	GA	Secretor	493461
f242	Finnish	Male	Vaginal	GA	Secretor	524289
f251	Finnish	Female	C section	GA	Secretor	421609
i01	Indian	Male	Vaginal	GA	Secretor	424473
i02	Indian	Female	Vaginal	AA	Non secretor	483961
i03	Indian	Male	Vaginal	GG	Secretor	322455
i04	Indian	Male	Vaginal	AA	Non secretor	208728
i06	Indian	Male	C section	GG	Secretor	201956
i07	Indian	Female	C section	GG	Secretor	318893
i08	Indian	Female	Vaginal	GG	Secretor	364666
i09	Indian	Female	Vaginal	GG	Secretor	229713
i10	Indian	Male	Vaginal	GA	Secretor	232140

i12	Indian	Female	Vaginal	GG	Secretor	405863
i16	Indian	Male	C section	GG	Secretor	275190
i18	Indian	Female	C section	GG	Secretor	221005
i20	Indian	Female	Vaginal	GA	Secretor	222782
i23	Indian	Male	Vaginal	GG	Secretor	246660
i24	Indian	Male	C section	GG	Secretor	265234
i25	Indian	Female	C section	GG	Secretor	205733
i28	Indian	Female	Vaginal	GA	Secretor	222471
i29	Indian	Female	Vaginal	GA	Secretor	221827
i30	Indian	Male	Vaginal	GG	Secretor	202441
i31	Indian	Female	Vaginal	AA	Non secretor	245464
i32	Indian	Female	Vaginal	AA	Non secretor	224905
i33	Indian	Female	Vaginal	GA	Secretor	271300
i34	Indian	Female	C section	GG	Secretor	230294
i39	Indian	Male	Vaginal	GG	Secretor	741699
i41	Indian	Male	Vaginal	GG	Secretor	610929
i43	Indian	Male	Vaginal	GG	Secretor	164579
i45	Indian	Male	Vaginal	GA	Secretor	199151
i46	Indian	Female	Vaginal	GG	Secretor	216431
i47	Indian	Male	C section	GA	Secretor	175229
i49	Indian	Male	Vaginal	GG	Secretor	575204
i50	Indian	Female	Vaginal	AA	Non secretor	208067
i51	Indian	Female	C section	GA	Secretor	198928
i52	Indian	Male	Vaginal	AA	Non secretor	407312
i53	Indian	Male	Vaginal	GA	Secretor	606248
i54	Indian	Male	Vaginal	GA	Secretor	485659
i55	Indian	Male	Vaginal	GA	Secretor	445029

i56	Indian	Male	C section	GA	Secretor	481824
i57	Indian	Female	C section	GA	Secretor	498550
i58	Indian	Female	C section	GA	Secretor	298966
i59	Indian	Male	C section	GG	Secretor	414983
i60	Indian	Male	Vaginal	GA	Secretor	462179
i61	Indian	Male	Vaginal	AA	Non secretor	670301

Supplementary Table S5. Details of birth mode, *FUT2* genotype and number of good quality sequences assembled per sample (f: Finnish, i: Indian). In total 83 samples were used for16S rRNA gene-based microbial profiling (Finnish: n=41 and Indian: n=42).
